# Relationship between Adiponectin Gene Polymorphisms and Late-Onset Alzheimer’s Disease

**DOI:** 10.1371/journal.pone.0125186

**Published:** 2015-04-22

**Authors:** Zhuling Yu, Wei Li, Deren Hou, Lin Zhou, Yanyao Deng, Mi Tian, Xialu Feng

**Affiliations:** 1 Department of Neurology, The Third Xiangya Hospital, Central South University, Changsha, China; 2 Department of Nerve medical center, The First Hospital of Changsha, Changsha, China; 3 Department of Geriatric Neurology, Xiangya Hospital, Central South University, Changsha, China; Cedars-Sinai Medical Center, Maxine-Dunitz Neurosurgical Institute, UNITED STATES

## Abstract

In recent years, researchers have found that adiponectin (ANP) plays an important role in the pathogenesis of Alzheimer's disease (AD), and low serum concentrations of ANP are associated with AD. Higher plasma ANP level have a protective effect against the development of cognitive decline, suggesting that ANP may affect AD onset. Meanwhile, accumulating evidence supports the crucial role of ANP in the pathogenesis of AD. To study the relationship between ANP gene polymorphisms (rs266729, -11377C>G and rs1501299, G276T) and late-onset AD (LOAD), we carried out a case-control study that included 201 LOAD patients and 257 healthy control subjects. Statistically significant differences were detected in the genotype and allelotype frequency distributions of rs266729 and rs1501299 between the LOAD group and the control group, with a noticeable increase in the G and T allelotype frequency distributions in the LOAD group (*P* < 0.05). Logistic regression analysis using recessive model and additive model revealed that the rs266729 GG and rs1501299 TT genotypes are associated with a greater risk of LOAD. Haplotype analysis identified four haplotypes: CG, CT, GG, and GT. The frequencies of the CT and GG haplotypes were not significantly different (*P* > 0.05) between the LOAD group and control group, whereas the CG and GT haplotypes were significantly different (*P* < 0.05), suggesting a negative correlation between the CG haplotype and LOAD onset (OR = 0.74, 95% CI = 0.57–0.96, *P* = 0.022), and a positive correlation between the GT haplotype and LOAD onset (OR = 2.29, 95% CI = 1.42–3.68, *P* = 0.005). Therefore, we speculated that the rs266729 and rs1501299 of ANP gene polymorphisms and the GT and CG haplotypes were associated with LOAD.

## Introduction

Adiponectin (ANP) is a protein specifically secreted by adipocytes. A study by Kamogawa *et al*. [1] examining 517 middle-aged and elderly community residents found that low serum ANP concentrations were related to Alzheimer’s disease (AD). Moreover, logistic regression analyses with confounding factors, including age and the abdominal subcutaneous fat area, showed that a 10 mg/l increase in plasma ANP had a protective effect against the development of mild cognitive impairment (MCI) in men, suggesting that ANP may affect AD onset. At present, studies [2–7] on the relationship between *ANP* gene polymorphisms and serum ANP concentrations, obesity, metabolic syndrome, insulin resistance, and type 2 diabetes have been performed, but none have reported the relationship between *ANP* gene polymorphisms and late-onset AD (LOAD). In this study, we used MALDI-TOF mass spectrometry to genetically type *ANP* gene loci, specifically the -11377C>G (rs266729) and G276T (rs1501299) polymorphisms, to determine the genotype and allele frequency distributions among LOAD subjects and healthy controls. In addition, haplotyping and multiple-factor logistic analyses were performed to determine the correlation between *ANP* gene polymorphisms (rs266729 and rs1501299) and LOAD, thereby enabling the examination of LOAD pathogenesis at the gene level and providing new information for the genetic diagnosis and early treatment of LOAD.

## Materials and Methods

### Study Subjects

Subjects in the LOAD group were AD patients confirmed by the Neurology Department of the Third Xiangya Hospital of Central South University, China. The control group comprised healthy people receiving physical check-ups at the Health Management Center at the same hospital. Clinical data and peripheral blood samples were collected from all of the subjects. There were 201 members in the LOAD group, including 90 males and 111 females with an average age of 76.79 ± 5.65 years. The disease course ranged from 1–15 years, with Mini Mental State Examination (MMSE) scores of 15.36 ± 3.48, activities of daily living (ADL) scores of 54.24 ± 7.82, and modified Hasegawa’s dementia scale (HDS-R) scores of 14.52 ± 4.26. Inclusion criteria for the LOAD group were: (1) patients confirm with AD diagnostic criteria in the revised fourth edition of the “Diagnostic and Statistical Manual for Mental Illness” (DSM IV-R), and of the US National Institute of Neurological and Communicative Disorders and Stroke and the Alzheimer’s Diseases and Related Disorders Associations (NINCDS-ADRDA);(2) patients confirm with minimental state examination (MMSE) scores for illiterate patients (≤17points), patients with primary education (≤20 points), and patients educated to high school and above (≤24 points). Additionally, Clinical Dementia Rating (CDR) scores ≥1 point, Hamilton Depression Scale (HAMD) scores ≤7 points, Hachinski Ischemic Score (HIS) <4points, activities of daily living (ADL) scores >20 points, and revised Hasegawa Dementia Scale (HDS-R) scores <15 points;and (3) age of onset ≥65 years. Exclusion criteria for the LOAD group were:(1)vascular dementia;(2) pseudo-dementia caused by depression;and (3) patients with myocardial infarction, heart failure, stroke, type 2 diabetes mellitus, and atherosclerosis. There were 257 members in the control group, including 121 males and 136 females with an average age of 75.88 ± 6.50 years. Inclusion criteria for the control group were: (1) normal intelligence and daily living abilities; and (2) patients confirm with MMSE scores for illiterate people (≥17 points), people with primary education (≥20 points), and people educated to high school and above (≥24 points). The controls were also confirmed not to have major diseases such as myocardial infarction, congestive heart failure, cerebral apoplexy, type 2 diabetes, atherosclerosis, and autoimmune diseases. All subjects were Hunan Han Chinese. The study was approved by the Ethics Committee of the Third Xiangya Hospital, and written informed consent was provided by the subjects or their relatives. We kept the subjects and their families informed about our study. There were no significant differences in age or gender between the LOAD group and control group.

### Genomic DNA extraction

DNA was extracted using whole-genome DNA kits (Dingguo Changsheng Biotechnology Co., Ltd., Beijing, China) and stored at -80°C.

### Primer design

Primers were designed using Assay Design 3.1 software from Sequenom (San Diego, CA, USA) and synthesized by Gene-Cloud Biotechnology Co., Ltd. (Beijing, China). PCR-specific and single-base extension primer sequences are provided in Table 1.

**Table 1:** SNP amplification and extension primer sequences.

dbSNP rs number	Marker	SNP	Primer sequence (5′→3′)	
rs266729	-11377C>G	C/G	forward primer	ACGTTGGATGACACCTTGGACTTTCTTGGC
			reverse primer	ACGTTGGATGATGTGTGGCTTGCAAGAACC
			extension primer	GCTCATGTTTTGTTTTTGAAG
rs1501299	G276T	G/T	forward primer	ACGTTGGATGCTTTGCTTTCTCCCTGTGTC
			reverse primer	ACGTTGGATGTCATCACAGACCTCCTACAC
			extension primer	ATAGGCCTTAGTTAATAATGAATG

SNP, single nucleotide polymorphism.

### SNP genotyping

The Sequenom MassARRAY system was used for genotyping genetic loci, and the analysis was performed as follows: 1) PCR amplification of DNA segments containing *ANP* loci. Purified genomic DNA samples were diluted to specific volumes, and 1 μl of each sample was added to specific wells of a 384-well plate. Next, 4 μl of the PCR amplification system was added, which contained Taq polymerase (1 U), genomic DNA (10 ng), PCR primers (0.1 μmol/l each), and dNTPs (500 μmol/l). PCR was performed at 94°C for 15 min, followed by 45 cycles of 20 s at 94°C, 30 s at 56°C, and 60 s at 72°C with a final extension step of 3 min at 72°C. 2) Shrimp alkaline phosphatase (SAP; 0.5 U) was added to eliminate residual dNTPs, and the reactions were incubated at 37°C for 40 min and then at 85°C for 5 min. 3) The samples were examined after PCR product purification and single base extension, and they were desalted by rinsing. Sample application and spectrometric detection were performed, and the experimental results were analyzed by TYPER 4.0 (Sequenom) to generate genotype data.

### Statistical Analysis

Statistical analysis was performed using SPSS 19.0 (IBM, Armonk, NY, USA). Comparisons of general characteristics between the two groups were performed using *t* or χ^2^ tests. Genotype and allele frequency comparisons between the two groups were performed using χ^2^ tests. SHEsis (http://analysis2.bio-x.cn/myAnalysis.php) was used to determine the Hardy-Weinberg equilibrium, odds ratios (OR), and 95% confidence intervals (CIs). Logistic regression analysis was used to examine the relationship between rs266729 and rs1501299 polymorphisms and LOAD susceptibility after adjusting for confounding factors. *P* < 0.05 was defined as statistically significant.

## Results

### Comparison of clinical characteristics

Clinical characteristics for all subjects are shown in Table 2. There were no significant differences between the LOAD group and control group in terms of gender, mean age, loss of spouse, body mass index (BMI), head trauma, hypertension, triglyceride (TG), low-density lipoprotein (LDL), high-density lipoprotein (HDL), or fasting blood glucose (*P* > 0.05). However, there were significant differences in the education level and total cholesterol (TC) (*P* < 0.05) between the two groups, indicating that a low level of education and high cholesterol levels were risk factors for AD.

**Table 2:** Comparison of clinical characteristics between the AD and control groups.

Index	AD group (*n* = 201)	Control group (*n* = 257)	*P*
Gender (male/female)	90/111	121/136	0.623
Mean age (years)	76.79 ± 5.65	75.88 ± 6.50	0.113
Education (≤ primary school/> primary school)	117/84	120/137	0.014*
Widowed (yes/no)	89/112	92/165	0.065
Head trauma (yes/no)	8/193	11/246	0.873
Hypertension (yes/no)	62/139	65/192	0.188
BMI (kg/m^2^)	22.65 ± 1.94	22.35 ± 1.48	0.068
TC (mmol/L)	5.12 ± 0.98	4.87 ± 0.69	0.002*
TG (mmol/L)	1.85 ± 1.12	1.98 ± 1.40	0.294
LDL (mmol/L)	2.60 ± 1. 09	2.46 ± 1.13	0.227
HDL (mmol/L)	1.32 ± 0.36	1.33 ± 0.77	0.722
Fasting blood glucose (mmol/L)	5.34 ± 0.90	5.20 ± 0.71	0.065

* *P* < 0.05. ≤ primary school, illiterate or educated to primary school level;> primary school, educated above primary school level; BMI, body mass index;TC, total cholesterol; TG, triglyceride; LDL, low-density lipoprotein; HDL, high-density lipoprotein.

### MassArray spectrometric genotype-feature peak charts

**Fig 1:**
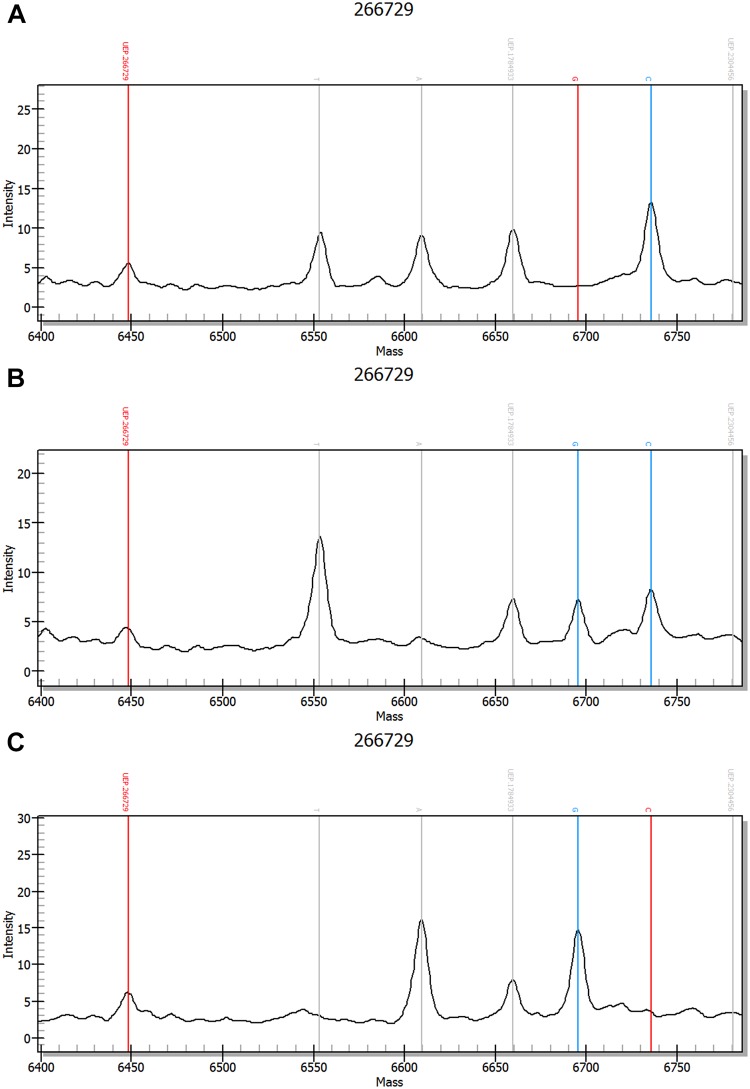
Spectrometric peak chart of rs266729. (A) *ANP* gene rs266729 (-11377C>G) CC genotype. (B) *ANP* gene rs266729 (-11377C>G) CG genotype. (C) *ANP* gene rs266729 (-11377C>G) GG genotype.

**Fig 2:**
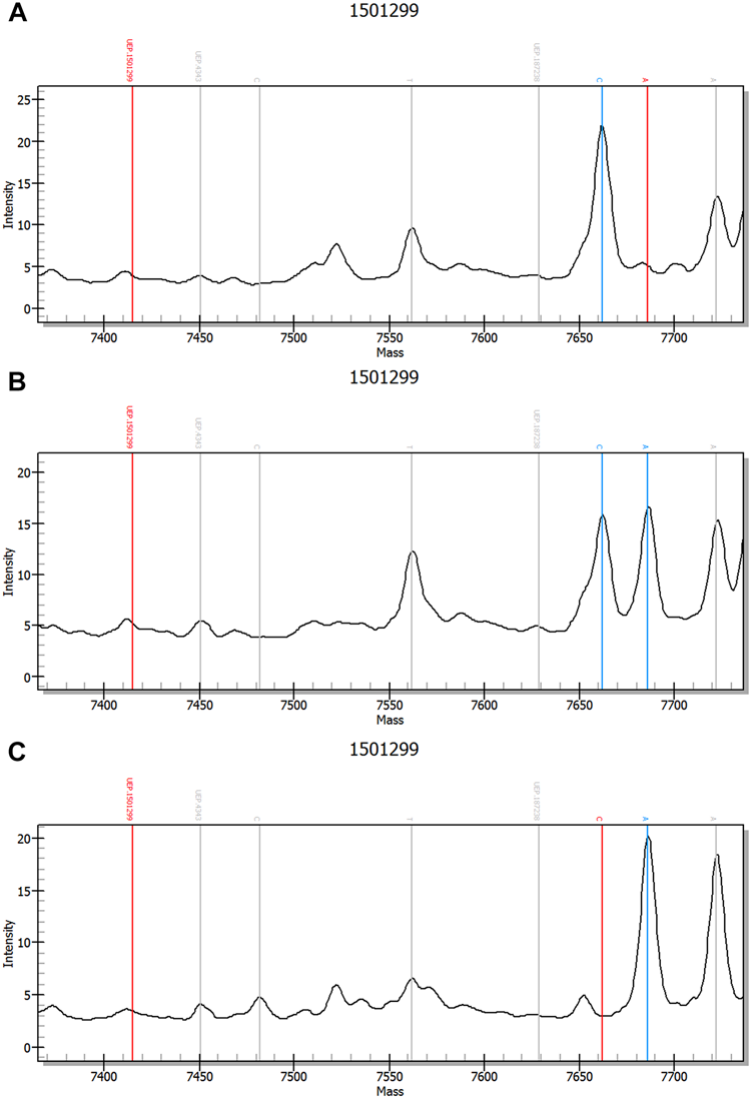
Spectrometric peak chart of rs1501299. (A) *ANP* gene rs1501299 (G276T) GG genotype. (B) *ANP* gene rs1501299 (G276T) GT genotype. (C) *ANP* gene rs1501299 (G276T) TT genotype.

In both groups, MassArray analysis detected CC, GG, and CG genotypes for rs266729 and GG, TT, and GT genotypes for rs1501299. Spectrometric genotype-feature peak charts for rs266729 and rs1501299 are presented in Figs 1 and 2.

### Hardy-Weinberg analysis

Genotype distributions of both *ANP* SNPs (rs266729 and rs1501299) in controls and AD cases were in accordance with Hardy-Weinberg equilibrium (*P* > 0.05) (Table 3).

**Table 3:** Hardy-Weinberg analysis of rs266729 and rs1501299.

	rs266729		rs1501299	
	χ^2^	*P*	χ^2^	*P*
AD group	1.92	0.17	1.85	0.17
Control group	0.003	0.99	0.05	0.82

*P* > 0.05 indicates that the cohorts are suitable for genetic analysis.

### Polymorphism distribution of rs266729 in the LOAD group and control group

Differences between genotype and allelotype frequencies for rs266729 in the LOAD group and control group were statistically significant, with a noticeable increase in the G allelotype frequency detected in the LOAD group (OR = 1.42, 95% CI = 1.06–1.89, *P* < 0.05) (Table 4).

**Table 4:** Polymorphism distribution of rs266729.

Group	Cases	Genotypes, cases (%)			Alleles, cases (%)	
		CC	GG	CG	G	C
AD group	201	95 (47)	26 (13)	80 (40)	132 (33)	270 (67)
Control group	257	142 (55)	17 (7)	98 (38)	132 (26)	382 (74)
χ^2^	6.3				5.3	
*P*	*P* = 0.04*				*P* = 0.018*	

* *P* < 0.05 indicates statistically significant differences.

### Polymorphism distribution of rs1501299 in LOAD group and control group

The differences between genotype and allelotype frequencies for rs1501299 in the LOAD group and control group were statistically significant, with a noticeable increase in the T allelotype frequency detected in the LOAD group (OR = 1.44, 95% CI = 1.07–1.94, *P* < 0.05) (Table 5).

**Table 5:** Polymorphism distribution of rs1501299.

Group	Cases	Genotypes, cases (%)			Alleles, cases (%)	
		GG	TT	GT	T	G
AD group	201	105 (52)	21 (10)	75 (37)	117 (29)	285 (71)
Control group	257	155 (60)	12 (5)	90 (35)	114 (22)	400 (78)
χ^2^	6.7				5.7	
*P*	0.035*				0.017*	

* *P* < 0.05 indicates statistically significant differences.

### Logistic regression analysis of rs266729 and rs1501299

After adjusting for confounding factors such as education, gender, age, BMI, and TC, multiple-factor conditional logistic regression analysis with a recessive model showed that the likelihood of rs266729 GG allelotype carriers developing AD was 1.82 times greater than that of the CG+CC genotype carriers, In additive model, the likelihood of rs266729 GG allelotype carriers developing AD was 2.01 times greater than that of the CG genotype carriers, and the GG allelotype was significantly related to the risk of LOAD onset (Table 6).

**Table 6:** Logistic regression analysis of rs266729.

rs266729		*P*	OR (95% CI)
Dominant	GG+CG vs. CC	0.13	1.37 (0.93–2.05)
Recessive	GG vs. CG+CC	0.036	1.82 (0.07–3.69)
Additive	GG vs. CC	0.078	1.96 (1.15–3.77)
	GG vs. CG	0.007	2.01 (1.05–4.30)

Adjusted for education, gender, age, TC, and BMI. OR, odds ratio; CI, confidence interval.

Similarly, for rs1501299, the likelihood of TT allelotype carriers developing AD was 2.05 times greater than that of GT+GG genotype carriers, In additive model, the likelihood of rs1501299 TT allelotype carriers developing AD was 2.58 times greater than that of the GG genotype carriers, and the TT allelotype was significantly related to the risk of LOAD onset (Table 7).

**Table 7:** Logistic regression analysis of rs1501299.

rs1501299		*P*	OR (95% CI)
Dominant	TT+GT vs. GG	0.12	1.79 (1.50–2.79)
Recessive	TT vs. GT+GG	0.017	2.05(0.82–4.66)
Additive	TT vs. GG	0.01	2.58 (1.22–5.48)
	TT vs. GT	0.065	2.3 (1.17–4.65)

Adjusted for education, gender, age, TC, and BMI. OR, odds ratio; CI, confidence interval.

### Construction and analysis of *ANP*-related SNP haplotypes

SHEsis software was used to perform linkage disequilibrium and haplotype analysis on rs1501299 and rs266729 of the *ANP* gene. With haplotype frequencies > 3%, D′ was 0.70 between rs1501299 and rs266729. There were four haplotypes (CG, CT, GG, and GT); of these, the CG haplotype was negatively correlated with AD onset (OR = 0.74, 95% CI = 0.57–0.96, *P* = 0.022), suggesting that individuals carrying this haplotype have a reduced risk of developing AD. Conversely, the GT haplotype was positively correlated with AD onset (OR = 2.29, 95% CI = 1.42–3.68, *P* = 0.005), suggesting that individuals carrying this haplotype are at a greater risk of developing AD (Table 8).

**Table 8:** Correlation analysis between *ANP* gene haplotypes and AD.

Haplotype	AD (freq)	Control (freq)	χ^2^	*P*	OR (95% CI)
CG	202.28 (0.503)	297.62 (0.579)	5.23	0.022*	0.74 (0.57–0.96)
CT	67.72 (0.168)	84.38 (0.164)	0.03	0.86	1.03 (0.73–1.46)
GG	82.72 (0.206)	102.38 (0.199)	0.06	0.81	1.04 (0.75–1.44)
GT	49.28 (0.123)	29.62 (0.058)	12.1	0.005*	2.29 (1.42–3.68)

* *P* < 0.05 indicates statistically significant differences.

## Discussion

ANP is one of the most abundant proteins secreted by adipose tissues. Moreover, the ANP receptor is widely expressed in the central nervous system and surrounding tissues, with extremely high expression in the hippocampus. ANP, in combination with central ANP receptors, may activate signaling channels in the central nervous system and therefore participate in the regulation and control of cerebral function [8]. Recently, research has shown that a high concentration of circulating ANP is protective in AD [9–11]. In addition, Masaki *et al*. [9] found that hippocampal volumes and ANP concentrations were positively correlated in type 2 diabetics. Comparing circulating ANP concentrations between MCI and AD patients and healthy individuals, Teixeira *et al*. [10] found that circulating ANP concentrations in MCI and AD patients were generally low, suggesting that low serum ANP concentrations are closely related to the pathological process of AD as well as to cognitive dysfunction. Moreover, the relationship among cholinesterase inhibitors, adipocyte factors, and AD was examined by Kálmán *et al*. [11]. These authors found significantly increased ANP in AD patients taking donepezil, providing further evidence that increased serum ANP levels are protective for AD patients. The functional mechanism of ANP in AD involves the following: reduced amyloid-β (Aβ) generation and accumulation [12], prevention of pathological and virulent changes caused by central Aβ [13], improvement in insulin resistance in the central nervous system [14], protection against and reduction of nerve cell apoptosis [9, 15–16], improvements in blood-brain barrier function[17], alterations in immuno-inflammatory responses[18], protection of vascular endothelial cells, prevention of vascular atherosclerosis, and regulation of glycolipid metabolism [19–20].

### Correlation analysis between the rs266729 polymorphism and LOAD

In the LOAD group, the G allele frequency was significantly higher than that in the control group (33% vs. 26%; *P* < 0.05). Moreover, there was a significant positive correlation between the rs266729 gene polymorphism and G allele frequency in the LOAD group (OR = 1.42). Therefore, the likelihood that patients carrying the G allele will develop LOAD is 1.42 times higher than the likelihood that patients without the G allele will develop LOAD. The G allele frequency significantly increases the LOAD onset risk, suggesting that the rs266729 polymorphism is associated with LOAD. Multiple-factor conditional logistic regression analysis using a recessive model (and correcting for confounding factors such as education, gender, age, BMI, and TC) revealed that the likelihood of GG allelotype carriers developing LOAD is 1.82 times higher than the likelihood of CG+CC genotype carriers developing LOAD which is 2.01 times higher than the likelihood of CG genotype carriers developing LOAD in additive model and is significantly related to LOAD onset risk. Recently, studies on the rs266729 polymorphism have found that CC allele carriers have low serum ANP concentrations[6], and GG gene carriers are more likely to suffer from metabolic syndrome and insulin resistance [3, 7], both of which are risk factors for AD onset. Consequently, the rise in LOAD onset risk for GG allele carriers may be caused by GG allele-induced reductions in ANP concentrations and increases in AD onset risk factors (e.g., metabolic syndrome and insulin resistance).

### Correlation analysis between the rs1501299 polymorphism and LOAD

The T allele frequency was significantly higher in the LOAD group than in the control group (29% vs. 22%; *P* < 0.05, OR = 1.42). The likelihood of T allele carriers developing LOAD is 1.44 times higher than the likelihood of those without the T allele developing LOAD, indicating that the T allele significantly increases the LOAD onset risk. Multiple-factor conditional logistic regression analysis using a recessive model (with correction for confounding factors) revealed that the likelihood of TT allelotype carriers developing LOAD is 2.05 times higher than the likelihood of those with the GT+GG genotype developing LOAD, which is 2.58 times higher than the likelihood of GG genotype carriers developing LOAD in additive model and the TT allelotype is significantly related to the LOAD onset risk. Recently, studies on the rs1501299 polymorphism found that TT allele carriers have low serum ANP concentrations [2] and are more prone to obesity, metabolic syndrome, and type 2 diabetes [2,4,5,7]. Low serum ANP concentrations predict a high AD onset risk and decreased cognitive function and memory, and both atherosclerosis and type 2 diabetes are risk factors for AD onset. Consequently, the increase in the LOAD onset risk for TT allele carriers may be caused by the ability of the TT allele to reduce ANP concentrations and therefore increase AD onset risk factors (e.g., obesity, metabolic syndrome, and insulin resistance).

### Correlation analysis between LOAD and rs266729 and rs1501299 linkage disequilibrium and haplotypes

We found no linkage disequilibrium (D′ = 0.07) between rs266729 and rs1501299 at the *ANP* genetic locus. A potential explanation for this result may be that these loci are located far away from one another [21] or that different ethnicities [22] and small sample sizes were included in this study. Haplotyping suggests that individuals carrying the CG haplotype are less likely to develop LOAD compared with those carrying the GT haplotype.

We are the first to perform an association study between the rs266729 and rs1501299 polymorphisms of the *ANP* genetic locus and LOAD. Moreover, we are the first to report that carriers of the G allele at the rs266729 locus and carriers of the T allele at the rs1501299 locus are more likely to develop LOAD. After correcting for confounding factors, logistic regression analysis using a recessive model revealed significant positive correlations between rs266729 and rs1501299 and LOAD onset. Haplotyping suggests that the CG and GT haplotypes are correlated with LOAD onset. Our findings provide a novel perspective regarding the genetic onset of LOAD. Because of the limited study population, regions, and sample size included in this study, our conclusions require verification using larger sample sizes with additional races and individuals from different geographical regions. According to current research on the rs266729 and rs1501299 gene polymorphisms, we predict that the association among the GG genotype of the rs266729 locus, the TT genotype of the rs1501299 locus, and the increased LOAD onset risk is potentially related to reduced ANP levels, providing a theoretical basis for further studies on the relationship between ANP and LOAD.
