# Correction: Relationship between Adiponectin Gene Polymorphisms and Late-Onset Alzheimer's Disease

**DOI:** 10.1371/journal.pone.0130521

**Published:** 2015-06-10

**Authors:** The PLOS ONE Staff

The authors are listed out of order. The publisher apologizes for this error. Please view the correct author order, affiliations, and citation here:

Wei Li^1^,^2☯^, Zhuling Yu^1☯^, Deren Hou^1^*, Lin Zhou^3^, Yanyao Deng^1^, Mi Tian^1^, Xialu Feng^1^

^1^Department of Neurology, The Third Xiangya Hospital, Central South University, Changsha, China ^2^Department of Nerve medical center, The First Hospital of Changsha, Changsha, China ^3^Department of Geriatric Neurology, Xiangya Hospital, Central South University, Changsha, China

^☯^These authors contributed equally to this work

Li W, Yu Z, Hou D, Zhou L, Deng Y, Tian M, et al. (2015) Relationship between Adiponectin Gene Polymorphisms and Late-Onset Alzheimer’s Disease. PLoS ONE 10(4): e0125186. doi:10.1371/journal.pone.0125186
